# Numerical Study on Tsunami Hazard Mitigation Using a Submerged Breakwater

**DOI:** 10.1155/2014/863202

**Published:** 2014-08-19

**Authors:** Taemin Ha, Jeseon Yoo, Sejong Han, Yong-Sik Cho

**Affiliations:** ^1^Coastal Disaster Research Center, Korea Institute of Ocean Science & Technology, 787 Haeanro, Ansan 426-744, Republic of Korea; ^2^Department of Civil and Environmental Engineering, Hanyang University, 222 Wangsimni-ro, Seongdong-gu, Seoul 133-791, Republic of Korea

## Abstract

Most coastal structures have been built in surf zones to protect coastal areas. In general, the transformation of waves in the surf zone is quite complicated and numerous hazards to coastal communities may be associated with such phenomena. Therefore, the behavior of waves in the surf zone should be carefully analyzed and predicted. Furthermore, an accurate analysis of deformed waves around coastal structures is directly related to the construction of economically sound and safe coastal structures because wave height plays an important role in determining the weight and shape of a levee body or armoring material. In this study, a numerical model using a large eddy simulation is employed to predict the runup heights of nonlinear waves that passed a submerged structure in the surf zone. Reduced runup heights are also predicted, and their characteristics in terms of wave reflection, transmission, and dissipation coefficients are investigated.

## 1. Introduction

Water waves experience bottom topography changes as they propagate from an offshore region to coastal areas. Furthermore, water waves are subjected to several variations in coastal areas due to the effects of coastal and offshore structures. In general, an accurate analysis of deformed waves around coastal structures, including refraction, diffraction, reflection, shoaling, and wave breaking effects, is directly related to the construction of economically sound and safe coastal structures, as wave characteristics play an important role in determining the length, weight, and shape of levee bodies or armoring materials. Most coastal structures have been built within surf zones in order to protect beaches or ports. Because wave breaking is one of the most critical wave behaviors, it is crucial that variations in wave characteristics in the surf zone, especially with regard to nonlinear and turbulent effects, are correctly predicted to avoid coastal hazards. Therefore, a number of researchers have conducted numerical and experimental studies over the past few decades in order to identify complex wave transformations in coastal areas.

The potential impact of coastal hazards has been strengthened by weather variations due to global warming or active tectonic movement. For example, in 2003, the powerful typhoon Maemi struck Republic of Korea and killed 117 people. Maemi subsequently caused $4.1 billion in damage, making it the worst typhoon ever to hit the Korean Peninsula. In 2011, the Tohoku tsunami brought destruction along the Pacific coastline of Japan's northern islands, causing almost 20,000 fatalities and 300 billion USD damage. The tsunami also propagated throughout the Pacific Ocean region, eventually reaching the entire Pacific coast of North and South America from Alaska to Chile. Due to the occurrence of such destructive phenomena, there has been an increasing demand for improving and reinforcing coastal structures against intensified coastal hazards.

One of the most effective solutions to improve the stability of coastal structures is to construct a submerged breakwater in front of at-risk infrastructure. The success of such a strategy is due to the mechanism of energy dissipation at the edges of a submerged breakwater, whereby waves are broken down into higher harmonics and wave energy is partially reflected. Furthermore, a submerged breakwater has the advantages of both engineering efficiency to protect coastal communities, and an eco-friendly design, which ensures that the natural landscape and ecosystem are preserved. As a result, extensive research on hazard mitigation using submerged structures has been conducted.

The majority of studies on submerged structures have been carried out in an attempt to understand the evolution of an incident wave during propagation over the submerged crest and examine the effect of waves transmitted to shoreline and coastal structures. The interaction between waves and submerged breakwaters under nonbreaking conditions has been thoroughly investigated using different approaches [1–3]. In the last decade, it has become popular to identify wave transformations using Navier-Stokes equations (NSE). Huang et al. [4] presented an analysis of solitary wave interactions with submerged rectangular permeable structures based on NSE, while Lin [5] considered turbulent processes in the equations and investigated solitary wave evolution, energy reflection, transmission, and dissipation. Due to simulation simplicity and similarities in wave hydrodynamics, solitary waves have been employed over the last few decades to study tsunami behavior [6, 7]. Tsunami-like trains, which eventually break near the shoreline, may form a sequence of turbulent bores propagating toward shallow water or alternatively collapse upon nearshore breakwaters, thereby generating an overtopping flow. Such violent breaking waves and their accompanying wave forces can cause different structural failure mechanisms [8]. However, the manner in which a submerged structure can influence the runup of solitary waves on a beach has rarely been studied, even though this information is directly related to the mitigation of coastal hazards.

In this study, the NEWTANK (numerical wave tank) model [9, 10], which is a well-validated numerical model using a large eddy simulation, is employed to predict runup heights of nonlinear waves that passed a submerged structure in the surf zone. Reduced runup heights are predicted and their characteristics in terms of wave reflection, transmission, and dissipation coefficients are investigated. For verification purposes, the model is first applied to benchmark experiments simulating the transformation of solitary waves around a submerged structure. The model is then employed to predict the runup heights of solitary waves on a sloping beach according to the dimensions of submerged structures. Finally, variations in the runup processes for different cases are investigated, and a correlation between runup heights and the dimensions of submerged structures is analyzed.

## 2. Materials and Methods

### 2.1. Mathematical Model

The motions of an incompressible flow can be described by the Navier-Stokes equations, which represent the conservation of mass and momentum per unit mass in a bounded domain:

(1) $$\begin{array}{c} \frac{\partial u_{i}}{\partial x_{i}}=0,\\ \frac{\partial u_{i}}{\partial t}+\frac{\partial u_{i}u_{j}}{\partial x_{j}}=-\frac{1}{\rho }\frac{\partial p}{\partial x_{i}}+g_{i}+\frac{1}{\rho }\frac{\partial \tau_{ij}}{\partial x_{j}}, \end{array}$$

where *i*, *j* = 1, 2, 3 for three-dimensional flows, *u*_*i*_ denotes the *i*th component of the velocity vector, *ρ* is the density, *p* is the pressure, *g*_*i*_ is the *i*th component of the gravitational acceleration, and *τ*_*ij*_ is the molecular viscous stress tensor.

A direct numerical simulation using the NSE for turbulent flows at a high Reynolds number is computationally too expensive. As an alternative, the large eddy simulation (LES) approach [11], which solves large-scale eddy motions according to the space-filtered NSE and models small-scale turbulent fluctuations, has become an attractive strategy.

In the LES approach, the top-hat space filter [12] is applied to the NSE and the resulting filtered equations of motion include

(2) $$\begin{array}{c} \frac{\partial \bar{u_{i}}}{\partial x_{i}}=0,\\ \frac{\partial \bar{u_{i}}}{\partial t}+\frac{\partial \bar{u_{i}u_{j}}}{\partial x_{j}}=-\frac{1}{\rho }\frac{\partial \bar{p}}{\partial x_{i}}+g_{i}+\frac{1}{\rho }\frac{\partial \bar{\tau_{ij}}}{\partial x_{j}}, \end{array}$$

where $\bar{p}$ denotes the filtered pressure and $\bar{u_{i}}$ represents the filtered velocity. Note that the viscous stress terms are modeled by the Smagorinsky SGS model [13]. In general, the Smagorinsky coefficient used the value of *C*_*s*_ ~ 0.2 under the isotopic turbulence condition. In the numerical model, we used a value of 0.15 to perform the turbulence simulation suggested by previous works for wave-current interaction with structures [9, 10].

### 2.2. Numerical Solver for the NSE

For the numerical model presented here, the governing equations were solved by the finite difference method on a staggered grid system. A two-step projection technique [14], which has been shown to be very robust, was employed. The forward time difference method was used to discretize the time derivative. The convection terms were then discretized by a combination of the central difference and upwind methods, while only the central difference approach was utilized to discretize the pressure gradient and stress gradient terms. The volume of fluid (VOF) method was adopted to track the free surface. Detailed descriptions of the various numerical techniques may be found in previous reports [9, 10].

## 3. Results and Discussion

The following sections describe the numerical experiments that were conducted to demonstrate the accuracy of the numerical model. The model generally solves the NSE and employs the VOF method to track free surface movement. However, the numerical scheme was modified to include a mass source function in the governing equations, while pressure-Poisson equations were utilized for solving [15]. A direct forcing immersed boundary method [15] was employed in numerical experiments to replace a solid body with immersed boundary forces and to investigate wave interactions with coastal structures. At the outgoing boundary, a sponge layer was used to absorb the wave energy. A damping term was added to each momentum equation to dissipate wave energies. Details regarding the numerical technique may be found in a previous report by Ha et al. [16].

### 3.1. Overflow without Waves at a Vertical Seawall

If the water level rises above the crest level of a structure, as may be the case during extreme storm surges, overflow occurs. That is, seawater flows over the crest of the seawall. Since such a scenario has been responsible for many seawall failures in the past, wave overtopping is considered one of the most important processes to consider when designing seawalls.

To validate the present NSE model, overflow without waves at a broad crested weir was first studied. In this case, the water level was above the crest level of the structure and, here, the freeboard, *R*_*c*_, was defined as the vertical distance between the mean water level and the seawall crest level. Chadwick and Morfett [17] proposed a formula for discharge over a broad crested weir as follows:

(3) $$\begin{array}{c} q_{\text{weir}}=1.705\times C_{d}{\left|R_{c}\right|}^{3/2}, \end{array}$$

where *R*_*c*_ is the overflow depth and *C*_*d*_ is the discharge coefficient. A number of empirical discharge formulas have been developed to incorporate the value of *C*_*d*_. Chadwick and Morfett [17] proposed the following relationship with the experimentally adjusted coefficient *C*_*F*_:

(4) $$\begin{array}{c} C_{d}=0.848C_{F},\\ C_{F}≅0.91+0.21\frac{R_{c}}{B_{L}}+0.24\left(\frac{R_{c}}{R_{c}-d_{s}}-0.35\right), \end{array}$$

where *B*_*L*_ represents the width of the weir and *d*_*s*_ is the weir height.

**Figure 1:**
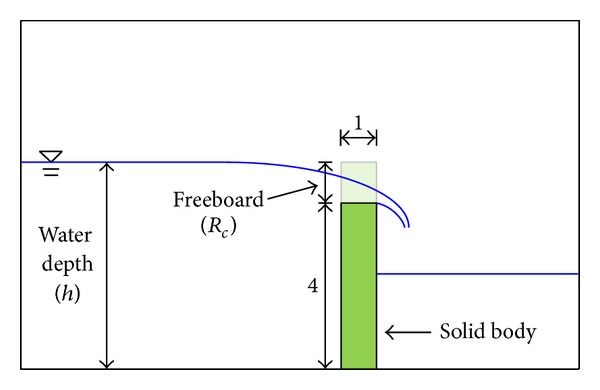
Sketch of the overflow at a vertical seawall.

Figure 1 showed a sketch of the overflow at a vertical seawall and model setup. In the numerical experiments, the initial water surface was first set up with a virtual embankment and, after setup, an embankment was removed in a moment to simulate overflow at a vertical seawall. Here, the water depth was 4.0 m, the width of the weir was 1.0 m, and the freeboard was varied in the range of 0.0 m to 0.8 m. A total of 500 cells were used in the *x*-axis direction with a uniform grid size of 0.2 m, while 60 cells were employed in the *z*-axis direction with a uniform grid size of 0.1 m. The time size was adjusted from approximately 0.001 s to 0.005 s at each time step using a stability criterion; the total simulation time was 30 s. For numerical stability condition, both the von Neumann method and the heuristic stability analysis were used to adjust a time step size at each time step. A solid boundary condition was applied at each side of the numerical tank to maintain the whole volume of fluid during computation.

**Figure 2:**
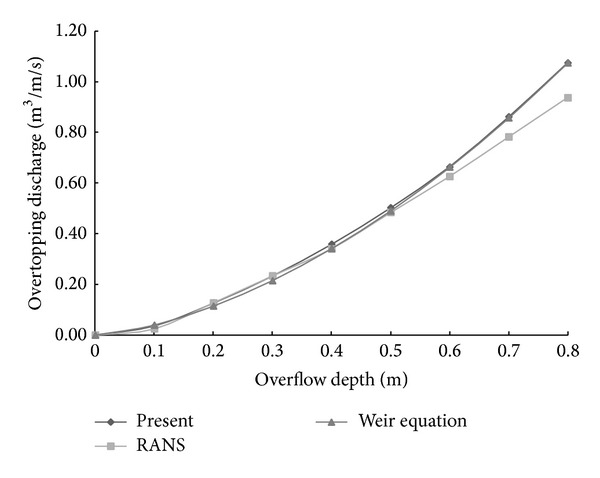
Comparison of the overtopping discharge rate obtained with the NSE models and the weir equations.

Table 1 and Figure 2 show comparisons of the overtopping discharge rate obtained with the weir equations and the NSE models. While the results from the present model are in good agreement with those acquired with the weir equations, the numerical model slightly overestimated the overtopping discharge when the total discharge was relatively small. It should be noted that this discrepancy may be caused by the viscous effects of the wall and could be neglected as the discharge increases. Soliman [18] conducted the same numerical tests with the Reynolds averaged Navier-Stokes equations (RANS) model developed by Lin and Liu [14]; the results are also compared with our findings in Table 1 and Figure 2. The present model showed slightly better agreement with the weir equations than the RANS model.

### 3.2. Transformation of Waves Propagating over a Submerged Structure

Water waves propagating over a submerged structure experience a number of complex processes, including nonlinear shoaling, an amplification of wave height, and the eventual initiation and termination of breaking as higher harmonics are released. Therefore, accurately predicting such transformations with numerical models is challenging. Beji and Battjes [1] carried out physical experiments to analyze the evolution of the frequency spectrum for waves propagating over a submerged structure. The subsequent findings have been widely used to verify a number of numerical models, including those of Lin and Li [19], Stelling and Zijlema [20], Roeber et al. [21], Ma et al. [22], and Tissier et al. [23]. A numerical model was applied to the aforementioned experiments to examine its viability for the case of waves propagating over a submerged structure. In general, it has been shown that a shoaling wave becomes nonlinear through the generation of bound higher harmonics on the upward slope. The generated higher harmonic waves are then released on the lee side of a submerged bar [24].

**Figure 3:**
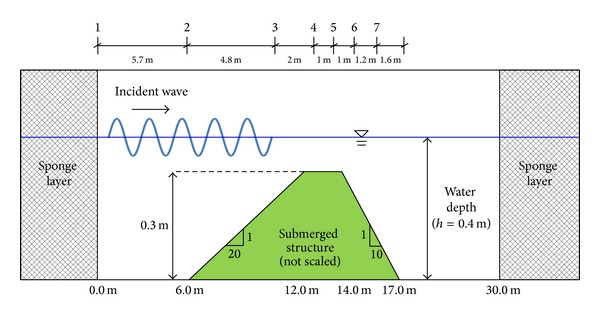
Sketch of the wave channel layout [1].

The model setup and bottom geometry are shown in Figure 3. In the model, the bottom geometry follows that used in the physical experiments of Beji and Battjes [1]. The length of the wave flume was 30.0 m with a water depth of *h* = 0.4 m. The submerged structure had a 1 : 20 upward slope and a 1 : 10 downward slope. The computational domain was 60.0 m long with 15.0 m of sponge layer at the left and right ends. A periodic incident wave with period *T* = 2.02 s and a wave height *H* = 0.02 m was generated using an internal wave maker, giving a relative wave height of *H*/*h* = 0.05 and relative wave depth of *kh* = 0.68, where *k* is the wavenumber. Following the convergence tests, the computational domain was discretized by horizontally uniform grids with a spacing of Δ*x* = 0.02 m and vertically nonuniform grids with Δ*z* = 0.002 ~ 0.01 m. The time step was automatically adjusted during the computations in order to satisfy the stability constraints.

**Figure 4:**
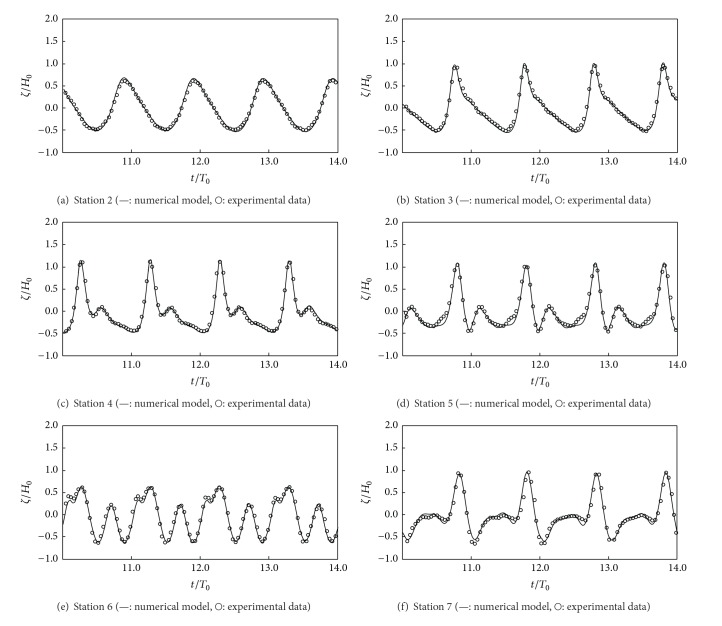
Water surface displacement obtained from the numerical model and experiments [1].

Figure 4 shows comparisons of the free surface elevation obtained from numerical results and experiments at six measurement locations. Water waves propagating into shallow water was steepened due to shoaling effects, and bound higher harmonics were subsequently generated by a nonlinear shoaling wave on the upward slope of the structure (Figures 4(a)–4(d)). The generated higher harmonic waves were released on the downward slope, resulting in an irregular wave pattern at stations 6 and 7, where wave dispersion was important. The numerical models generally yielded good predictions of the free surface evolution at these stations, indicating that the model was capable of simulating wave shoaling and dispersion over an uneven bottom geometry.

### 3.3. Runup and Breaking of Solitary Waves

The numerical model used in this study was subjected to a series of rigorous tests. For nonbreaking runup on a beach, the model was employed to simulate the runup and rundown of solitary waves on a beach with a steep slope [25]. The numerical results were then compared to experimental data in terms of the evolution of a free surface profile, and good agreement between the findings was obtained [15]. The model was later utilized to study wave interaction with a composite structure, and favorable agreement between the experimental and simulated results was again observed. For solitary wave interactions with structures, Synolakis [26] conducted experiments for an incident solitary wave propagating and breaking over a planar beach with a slope of 1 : 19.85. The simulation results obtained with the present model showed excellent agreement with the measurements. Furthermore, the model was employed to study the runup of solitary waves on a circular island [27], and the numerical results were consistent with the experimental data in terms of the evolution of the free surface profile and the velocity distribution around a circular island [15].

### 3.4. Tsunami Hazard Mitigation Using a Submerged Breakwater

Lin [5] conducted numerical experiments to study solitary wave interactions with submerged structures. The characteristics of wave transformations in terms of wave reflection, transmission, and dissipation (RTD) coefficients were investigated for various combinations of structure length *a* and height *b*. Lin [5] employed the incident energy flux, EF_inc_, the transformed energy flux, EF_trans⁡_, and the reflected energy flux, EF_ref_, to derive the RTD coefficients. The RTD coefficients were defined as the wave reflection, transmission, and dissipation coefficients (*K*_*R*_, *K*_*T*_, *K*_*D*_), respectively [5], and those were derived using the conservation of energy principle as follows:

(5) $$\begin{array}{c} K_{R}=\sqrt{\frac{-\text{E}{\text{F}}_{\text{ref}}}{\text{E}{\text{F}}_{\text{inc}}}},\\ K_{T}=\sqrt{\frac{\text{E}{\text{F}}_{\mathrm{trans}}}{\text{E}{\text{F}}_{\text{inc}}}},\\ K_{D}=\sqrt{\frac{TD}{\text{E}{\text{F}}_{\text{inc}}}}=\sqrt{1-K_{R}^{2}-K_{T}^{2}}. \end{array}$$

Here, the NSE model was employed to investigate how a submerged structure reduced tsunami-like solitary wave runup on an inclined beach based on the previous study.

**Figure 5:**
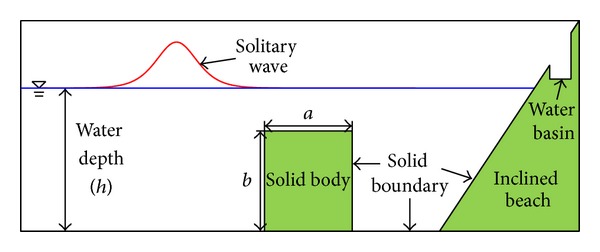
Illustration of a numerical wave tank.

In the numerical experiments, a submerged structure was constructed in front of an inclined beach in the numerical domain.  Figure 5 shows a schematic diagram of the problem setup. A numerical wave tank with a length of 110 m and a width of 0.5 m was employed in the simulation; the water depth was *h* = 1.0 m. The rectangular structure had the length *a* that varied from 1.0 m to 50 m and a height *b* that varied from 0.0 m to 0.9 m. The left edge of each different breakwater was located at *x* = 30.0 m. An incident solitary wave had a wave height of *H* = 0.1 m, which fixed the ratio of *H*/*h* = 0.1. In general, tsunamis in the ocean have very small wave heights when compared to the water depth and wavelength. Thus, it is rational to select a ratio of *H*/*h* = 0.1 when considering tsunami hazards. Additional simulations using *H*/*h* = 0.3 were conducted for a particular case to identify nonlinear effects on wave transformations. A uniform grid system of Δ*x* = Δ*y* = 0.05 m and Δ*z* = 0.01 m was selected following the results of convergence tests. Lin [5] deployed two numerical gauges at *x* = 1.0 m and *x* = 99.0 m to pick up wave signals for a calculation of wave RTD coefficients. In the numerical domain, an inclined beach with constant slope was employed at the right boundary so that the RTD coefficients derived by Lin (2004) could be utilized. Surely, the energy fluxes induced by turbulent flows at right side of the effective control surface (*x* = 99.0 m) for the solitary waves may become significant and bring a difficulty in computing RTD coefficients. Therefore, we adopted Lin's coefficients instead to investigate how transformed energy fluxes by a submerged structure generated runup on an inclined beach. This is quite reasonable because selection of appropriate dimension for the structure is directly related to an effective design of a submerged breakwater. Many engineers have been attracted to establishing effective design criteria for construction of a breakwater since it usually costs a lot of money. In this study, numerical experiments were conducted to understand how runup process on an inclined beach developed after an incident wave passed over the structure. As a result, we could evaluate qualitatively the structure as an effective mitigation method against a tsunami. In general, runup on an inclined beach becomes higher as the incident wave energy increases. We focused on discrepancy between this general criterion and numerical results.

To investigate solitary wave runup related to the RTD coefficients on an inclined beach, the runup coefficient was defined as follows:

(6) $$\begin{array}{c} R=\frac{R_{\max}}{R_{0}}, \end{array}$$

where *R*_max⁡_ denotes the maximum runup height on an inclined beach for each case and *R*_0_ is the maximum runup height computed without a submerged structure. The manner in which a submerged structure affected the runup behavior of solitary waves could be identified because the runup coefficient directly represented a variation of the runup heights.

**Figure 6:**
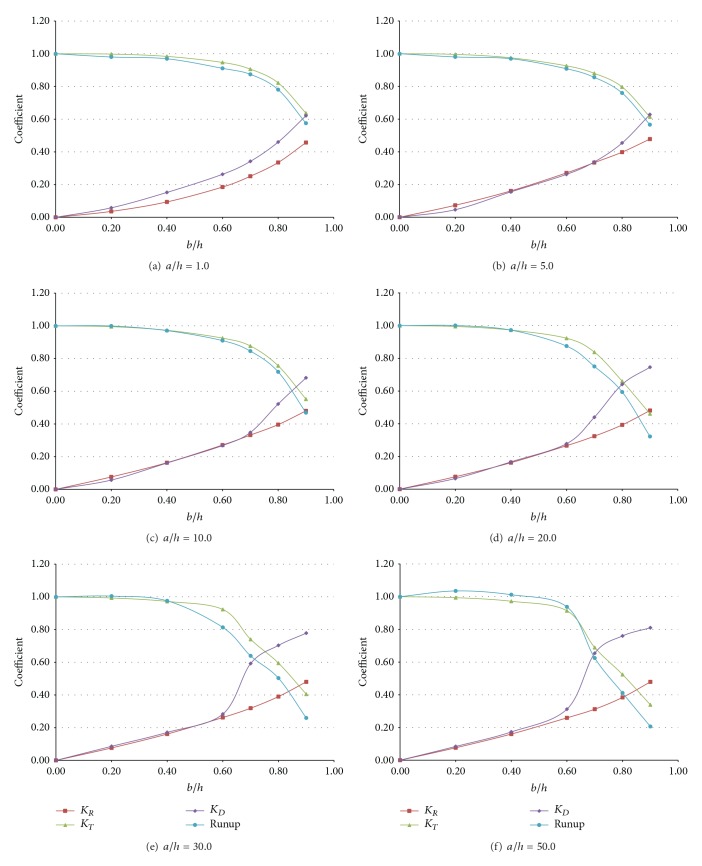
Variation of the RTD coefficients for different submerged structures (*H*/*h* = 1.0).

**Figure 7:**
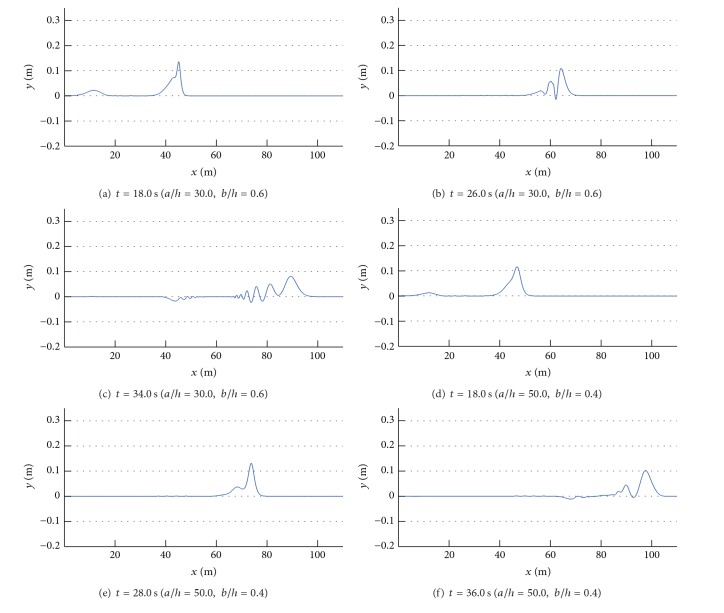
Fission process triggered by a submerged structure.

Figure 6 and Table 2 show the RTD coefficients depending on the horizontal and vertical length of a submerged structure. An inclined beach with a steep slope of 1/5 was initially employed since it is very difficult to analyze the results according to different sizes of submerged structures when wave breaking was induced during the runup. As shown in Figures 6(a)–6(c), the maximum runup height of a solitary wave was proportional to the transmission coefficient, but independent of the reflection or turbulent dissipation coefficient when the horizontal length of the structure was relatively short. Lin [5] identified that the solid front of a submerged structure generated upward motion of fluid particles while the incident wave propagated over the structure, and part of wave energy was reflected back due to the upward motion. Subsequently, the remainder of wave energy was transmitted over a submerged structure and would split into a number of individual solitons if the structure was sufficiently long (Figure 7). These phenomena were well represented by the variation in the runup coefficient. In the numerical results, the fission process usually decreased runup due to the dispersion of wave energy except some specific cases (*a*/*h* = 50.0) and the discrepancy would be discussed in detail later.

**Figure 8:**
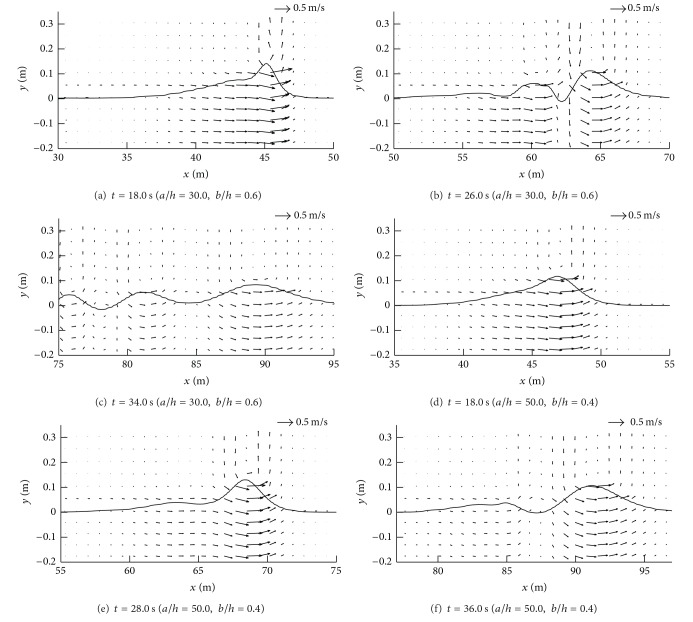
Variation in velocity distributions for solitary waves over a submerged structure.

In Figures 6(a)–6(c), the maximum runup height was independent of the horizontal length of the structure since the dispersion of a solitary wave was insignificant. In Figures 6(d)–6(f), the maximum runup height was proportional to the transmission coefficient when the vertical length of the structure was less than 0.4*h*. However, when the vertical length exceeded 0.6*h*, the maximum runup height was significantly reduced when compared to that of the transmission coefficient. It is thought that the runup process was influenced by the increased horizontal length of the structure and the maximum runup height decreased since the fission process took over for the structure that generated a number of solitons with decaying amplitude (Figures 7(a)–7(c)) as well as water particle velocities (Figures 8(a)–8(c)). The decelerated water particles implied less impact to an inclined beach, which resulted in less wave force and runup. Furthermore, the energy dissipation coefficient increased over the same range due to breaking of the solitary wave above a submerged structure, and the dissipation in wave energy due to the presence of a submerged structure also lowered the maximum runup height.

On the other hand, an interesting observation was noted for the case of *a*/*h* = 50.0. As shown in Figure 6(f), the maximum runup height was amplified and higher than that of the case without a structure when the vertical length of the structure was less than 0.6*h*. Not only was this feature different from the runup processes of the other cases, but also it was an unexpected result because a submerged structure was generally employed to dissipate wave energy and then lower the runup height. An increase in the horizontal length of the structure resulted in the fission of a solitary wave as well as shoaling. This shoaling effect would increase the runup height if the horizontal length exceeded some critical value, at which point shoaling was quantitatively dominant over the fission process. In the case of *a*/*h* = 50.0, fission took place over the submerged structure and a number of solitons with decaying amplitude were generated. However, the amplitudes of these solitons were augmented and upward components of water particle velocities were accelerated during propagation over the structure (Figures 7(d)–7(f) and 8(d)–8(f)), and breaking was eventually triggered when these solitons climbed up an inclined beach. When a solitary wave broke on an inclined beach, wave reflection did not occur during the runup and the maximum runup height was usually higher than that of a nonbreaking solitary wave. In particular, breaking on an inclined beach generated breaking-induced currents, which forced water waves to keep climbing up until they reached the highest level. Consequently, the broken waves increased the maximum runup height on an inclined beach, as depicted in Figure 6(f). When the vertical length of a submerged structure was higher than 0.7*h*, however, an incident wave was broken by the structure and runup coefficients rapidly decreased as the energy dissipation coefficients increased. During propagation over the structure, the dispersion of wave energy also occurred due to the fission process and runup coefficients decreased steeply compared to increases of the energy dissipation coefficients.

**Figure 9:**
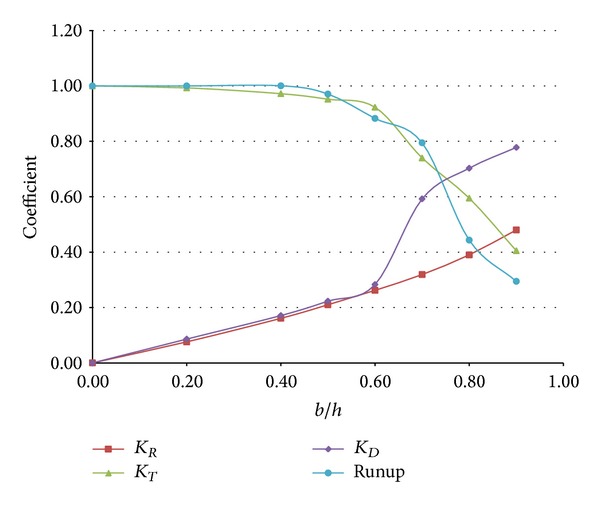
Variation of the coefficients on an inclined beach with a mild slope (*s* = 1/20).

Figure 9 shows the variation of the coefficients when breaking of a solitary wave occurred during runup on an inclined beach. An inclined beach with a mild slope of 1/20 was employed at the right boundary to trigger breaking on an inclined beach, while the horizontal length of the structure was fixed at *a*/*h* = 30.0 so that energy dissipation and retrenchment of the runup heights due to the submerged structure appeared. Since breaking of a solitary wave occurred regardless of the vertical length of the structure, the runup coefficient was constant at 1.0 when the vertical length ratio was small; that is, *b*/*h* ≤ 0.4. However, retrenchment of the runup coefficient ensued due to energy dissipation when the vertical length ratio was *b*/*h* > 0.4, and this phenomenon was accelerated in proportion to the transmission coefficient when *b*/*h* ≥ 0.6. From a qualitative standpoint, the numerical results shown in Figure 9 are similar to those displayed in Figure 6, but retrenchment of the runup coefficient started slightly later and varied more steeply in the former. It is thought that a breaking-induced current was quantitatively dominant over the fission process and energy dissipation occurred due to the presence of a submerged structure until the vertical length ratio was *b*/*h* ≤ 0.7, where the current forced the runup height to be increased. When *b*/*h* > 0.7, the runup coefficient exhibited a sharp decrease since fission and energy dissipation were dominant, but a mild slope still counteracted these diminishing factors. Thus, the decrease in the slope of the runup coefficient was less severe when compared to that of the transmission coefficient.

**Figure 10:**
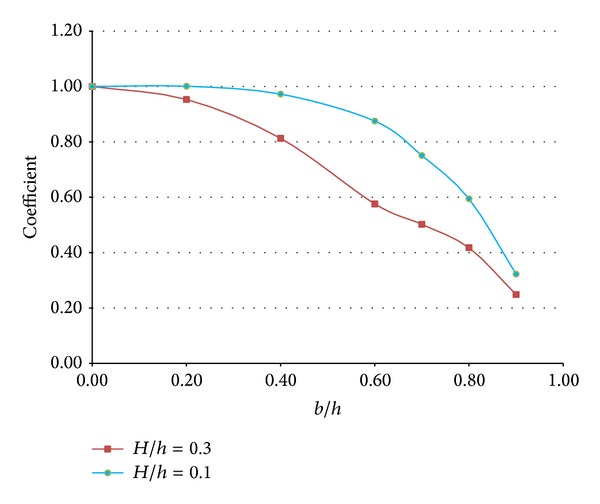
Variation of the runup coefficient with different wave heights.

Figure 10 shows the variation in the runup coefficient for incident solitary waves with different wave heights. In this case, an inclined beach with a steep slope of 1/5 was employed at the right boundary to prevent breaking on an inclined beach; the horizontal length of a structure was again fixed at *a*/*h* = 30.0 to allow for a comparison with the previous cases. Based on the behavior of the dissipation coefficient, Lin [5] roughly classified the steps into three types, namely, (1) a low submerged structure on which energy dissipation was insignificant, (2) a high structure on which vortex shedding and wave breaking dominated, and (3) a surface-piercing structure at which the energy dissipation coefficient decayed almost linearly with an increase in the structural height. In this study, a surface-piercing structure was not considered. The classification was characterized by the magnitude of *b*/*h* relative to *H*/*h* and, thus, a “low submerged structure” to a weakly nonlinear wave might become a “high structure” to a fully nonlinear wave. In Figure 10, this was well demonstrated at *b*/*h* = 0.2 and *b*/*h* = 0.4, where energy dissipation due to a submerged structure was insignificant for a wave with *H*/*h* = 0.1 but dominant for the wave with *H*/*h* = 0.3. Thus, relatively low structures could be served as high structures in highly nonlinear cases and this should be considered in designing a submerged breakwater against a tsunami. Since these highly nonlinear cases include strong turbulent flows and complex hydrodynamics, additional researches, such as physical experiments and three-dimensional numerical experiments with high resolution, are required to be conducted to validate this qualitative analysis and subsequently come up with an appropriate practical formula. The runup coefficients were calculated using relation between the maximum runup height on an inclined beach without a submerged structure and those with submerged structures. Thus, the runup coefficients were usually less than 1.0. Surely, the maximum runup height quantitatively became larger when an incident wave height increased.

## 4. Conclusion

In this study, a three-dimensional numerical model was employed to predict the runup heights of nonlinear waves that passed a submerged structure in the surf zone. Reduced runup heights were predicted and their characteristics in terms of wave reflection, transmission, and dissipation coefficients were investigated. The numerical results showed that a submerged structure could be useful for tsunami hazard mitigation when an appropriate design is implemented. While the model could qualitatively analyze a reduced pattern of runup heights, many processes remain unidentified. For example, the numerical data were insufficient to quantitatively analyze runup processes on a sloping beach despite the multitude of simulations conducted. It is noted that additional numerical and physical experiments are required to analyze the variation in runup processes caused by the dimensions of submerged structures. Furthermore, analytic and mathematical approaches are needed in order to identify proper design criteria for tsunami hazard mitigation.

## Figures and Tables

**Table 1:** Rates of overtopping discharges for different freeboards.

Tests	*R*_*c*_ [m]	Weir equation [m^3^/m/sec]	RANS model (Soliman, 2003 [18]) [m^3^/m/sec]	Present model [m^3^/m/sec]
W1	0.0	0.00000	0.00000	0.00000
W2	0.1	0.03900	0.02400	0.03566
W3	0.2	0.11400	0.12700	0.12559
W4	0.3	0.21500	0.23300	0.23254
W5	0.4	0.34100	0.34000	0.35765
W6	0.5	0.49000	0.48300	0.50152
W7	0.6	0.66200	0.62600	0.66406
W8	0.7	0.85700	0.78200	0.86093
W9	0.8	1.07500	0.93800	1.07532

**Table 2:** The runup coefficient for different combination of *a*/*h* and *b*/*h* (*H*/*h* = 0.1).

	*a*/*h* = 1.0	*a*/*h* = 5.0	*a*/*h* = 10.0	*a*/*h* = 20.0	*a*/*h* = 30.0	*a*/*h* = 50.0
*b*/*h* = 0.0	1.00000	1.00000	1.00000	1.00000	1.00000	1.00000
*b*/*h* = 0.2	0.98031	0.98031	0.99913	1.00097	1.00440	1.03517
*b*/*h* = 0.4	0.96935	0.96935	0.97097	0.97260	0.97553	1.01195
*b*/*h* = 0.6	0.91089	0.90818	0.90971	0.87512	0.81301	0.93777
*b*/*h* = 0.7	0.87422	0.85559	0.84544	0.75059	0.63904	0.62478
*b*/*h* = 0.8	0.78071	0.75974	0.71882	0.59419	0.50287	0.41111
*b*/*h* = 0.9	0.57519	0.56604	0.46876	0.32219	0.31561	0.30000
